# Public health round-up

**DOI:** 10.2471/BLT.25.010225

**Published:** 2025-02-01

**The Syrian Arab Republic upended**

Children arriving at a reception centre in Ar-Raqqah city, Syrian Arab Republic, having fled the escalating violence in Aleppo after the government’s overthrow. Since November 2024, some 882 000 people have been displaced as a result of unrest, with major implications for the health and well-being of the population.

**Figure:**
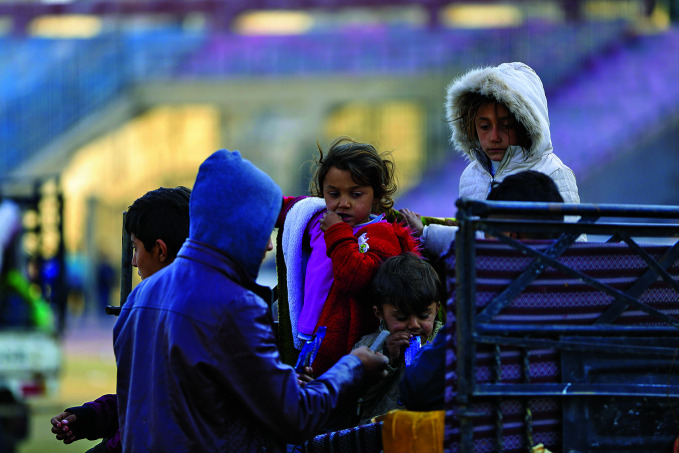
UNICEF/Muhannad Aldhaher

## Health under threat in the Syrian Arab Republic

The Syrian Arab Republic entered a period of turmoil, following the overthrow of the government of Bashar al-Assad in November 2024.

According to a 24 December report published by the World Health Organization (WHO), since November, intensified hostilities had displaced over 882 000 people, further disrupted access to health care and placed immense pressure on Syria's already fragile health system.

Attacks on health facilities were reported to have surged, with 37 WHO-confirmed incidents in the month prior to the 24 December report alone. Those attacks severely damaged health infrastructure, rendered ambulances non-operational and hindered access to lifesaving care. Even before the most recent developments, 141 health facilities in Idlib and northern Aleppo had been facing the risk of closure due to funding shortfalls.

The WHO response includes the delivery of 50 tonnes of lifesaving medical supplies via the European Union (EU) Humanitarian Air Bridge, an ad hoc initiative initially set up by the European Union Commission as part of the EU’s global coronavirus disease 2019 (COVID-19) response. The air bridge has since been used to transport aid to some of the world’s most fragile countries.

On 24 December, WHO launched a 56.4 million United States dollars (US$) flash appeal to support emergency response efforts. WHO also drafted a six-month strategy to address the immediate health needs emerging from the ongoing crisis, while engaging with the United Nations (UN) system’s planning efforts and the preparation of a Humanitarian Response Plan.

Over the next six months, WHO, together with its partners, will continue to focus on trauma care, continuity of essential services, disease outbreak prevention, patient referrals and strengthening health system coordination.

https://bit.ly/3Pmg8Lt

https://bit.ly/401Rogp

## Gaza’s health facility put out of action

Kamal Adwan Hospital was attacked on 27 December. According to a 28 December WHO media release, the hospital was severely damaged during the raid, putting it out of commission.

According to the WHO statement, in the week prior to the 27 December attack, bombardments in the vicinity of the hospital were reported to have killed 50 people, including five health workers from the hospital itself.

Since October 2023, WHO has repeatedly issued calls to protect health workers and hospitals as per international humanitarian law.

In related news, WHO Director-General Tedros Adhanom Ghebreyesus called for evacuations of patients from Gaza to be accelerated. In a 2 January 2025 statement posted on X, he noted that at the current rate of evacuation it would take 5–10 years to evacuate patients assessed to be critically ill, including thousands of children.

https://bit.ly/3WbeZdl

https://bit.ly/3WvEO8n

## Meeting trauma needs in Lebanon

In the aftermath of Israeli attacks on Lebanon, where a ceasefire was initiated on 27 November, thousands of civilians require reconstructive surgery and physical rehabilitation.

According to a 20 December WHO media release, as of that date more than 4 000 people had been killed and 17 000 injured in the country, with one in four sustaining life-changing injuries that will need long-term rehabilitation and, in some cases, assistive technologies and prosthetics.

"This need for specialized health care will persist for months and years to come,” said WHO Representative to Lebanon, Dr Abdinasir Abubakar, adding that Lebanon needs reconstructive surgeons to treat the severely injured, eye doctors to treat the thousands of people injured in the pager attacks that occurred on 17 and 18 September 2024, physiotherapists to start rehabilitating amputees and prosthetists to assist users of assistive devices.

https://bit.ly/4a4Mhk6

## Cholera in Yemen

Yemen has become the country with the highest burden of cholera globally, with persistent outbreaks since 2017, including the largest cholera epidemic in recent history.

According to a 23 December 2024 WHO media release, as of 1 December 2024, Yemen had reported 249 900 suspected cholera cases and 861 deaths, representing 35% of global cases and 18% of global mortality.

Contributing factors to the epidemic include poor access to safe drinking water, inadequate hygiene and limited health-care services. However, severe funding shortages have also hindered efforts to address the outbreak, notably leading to the closure of diarrhoea treatment centres and oral rehydration centres.

Working in collaboration with Yemen’s health ministry, WHO has implemented measures that include rapid response missions, laboratory support, and an oral cholera vaccination campaign that reached 3.2 million people.

https://bit.ly/4h2mX0y

## Attack on Sana'a airport, Yemen

Sana'a airport in Yemen was attacked on 26 December 2024 as members of a joint UN–WHO delegation, including Director-General Tedros Adhanom Ghebreyesus, were about to board a flight.

The WHO team had been in the country to negotiate the release of the 17 UN staff being detained and to assess the health and humanitarian situation in the country.

In a statement released three hours after the incident, the Director-General stated that the airport came under aerial bombardment. One of the plane’s crew members was injured. At least two people were reported to have been killed at the airport.

https://bit.ly/3BYPWn6

## Government spending on health down

Average per capita government spending on health in all country income groups fell in 2022 after a surge in the early years of the COVID-19 pandemic. This is according to the *2024 Global health expenditure report* published by WHO on 12 December.

The report indicated that after surging early in the pandemic, aggregate global health spending fell in 2022, to US$ 9.8 trillion, or 9.9% of global gross domestic product (GDP). This is the first decline in global health spending in real terms since 2000.

WHO Director-General Tedros Adhanom Ghebreyesus, underlined the importance of maintaining government spending on health to deliver universal health coverage, adding, “While access to health services has been improving globally, using those services is driving more and more people into financial hardship or poverty.” 

https://bit.ly/4j1YKcp

**Cover photo**

A team of health workers visits a nomad family to provide health-care services using remote digital technology, Mongolia.

**Figure:**
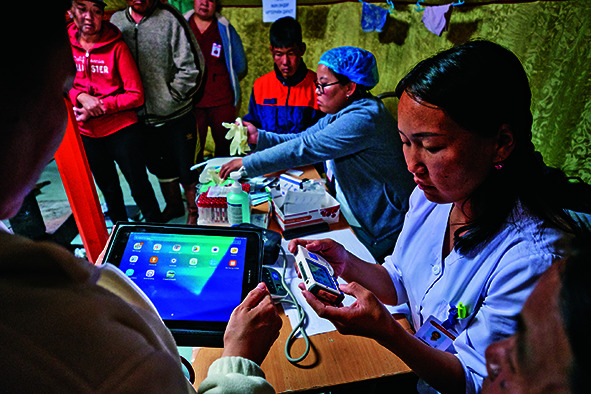
(WHO/Yoshi Shimizu)

## Uneven progress on drowning deaths

There has been an estimated 38% drop in the global drowning death rate since 2000, from an estimated 6.1 per 100 000 of population to 3.8 per 100 000 population currently – a major public health achievement.

However, according to WHO’s inaugural report on drowning prevention, which was published on 13 December 2024, progress in reducing drowning has been uneven.

For example, the WHO European Region saw a 68% fall in the drowning death rate between 2000 and 2021, compared to a 3% decline in the WHO African Region, which has the highest rate of any region with 5.6 deaths per 100 000 people.

One possible explanation for this disparity may be the levels of national commitments to address the issue: within the African Region, only 15% of countries had a national strategy or plan for drowning prevention, compared to 45% of countries in the European Region.

https://bit.ly/4j4agUE

## WHO Academy opens

The WHO Academy officially opened its doors in Lyon, France. Inaugurated on 18 December 2024 in a ceremony attended by WHO Director-General Tedros Adhanom Ghebreyesus, French President Emmanuel Macron, and numerous health ministers, international representatives, donors, and local French partners, the Academy will provide health professionals, policy-makers and WHO staff with critical skills, up-to-date knowledge, and expertise in public health.

The global training hub aims to enhance workforce development through in-person training sessions in Lyon and an online platform that is accessible worldwide.

“The WHO Academy will be game-changing, a first-of-its-kind global health learning centre that will equip health and care workers, policy-makers and our own global workforce with the competencies and skills they need to transform health systems and deliver health for all," said Director-General Tedros.

https://bit.ly/40lL04Q

**Looking ahead**

4 February. World Cancer Day: United by Unique. Events worldwide. https://bit.ly/3DIRYs5

13–15 February. Fourth Global NCD Alliance Forum. Kigali, Rwanda. https://bit.ly/4gUkBAK

18–20 February. Fourth Global Ministerial Conference on Road Safety. Marrakech, Morocco. https://bit.ly/3W48fxS

